# Guachamaca Extract

**Published:** 1882-10

**Authors:** 


### GUACHAMACA EXTRACT.

The *Medicinische Central-Zeitung* of July 15th, 1882, publishes the result of experiments instituted by Dr. Schiffer, of Berlin, for the purpose of determining the therapeutic value of the *extract of guachamaca*, a tree indigenous on the Apuche mountains. As a specific for all spastic conditions of the motor apparatus we so far possess, in reality, one remedy only, curare, which, however, on account of its danger, and the uncertainty of its action, as well as of the remedy itself, has mostly been used for the physiological experiments alone. Guachamaca extract, prepared from the bark of the quebracho plant, which belongs to the same class as the oleander (Nerium ol., L.), and of which latter the skin between tree and bark is also poisonous, is a remedy which, while not so dangerous in its effects, is far more reliable and uniform in its action, and can besides easily be procured pure and genuine, and of always equal strength, as it is by no means rare.

Schiffer experimented with this drug, and made his observations in the clinic of Professor Frerichs. He employed the remedy in solution, in the dose of one-sixth of a grain, and by the hypodermic method, in cases of tonic and clonic spasms of the muscular system, and always with a very good effect. He noted also that, administered internally, no matter in what solution, the drug was as little absorbed by the mucous membrane of the alimentary canal as curare is.—*Philadelphia Reporter.*

